# MMPs and angiogenesis affect the metastatic potential of a human vulvar leiomyosarcoma cell line

**DOI:** 10.1111/jcmm.12565

**Published:** 2015-05-22

**Authors:** Carlotta Alias, Laura Rocchi, Domenico Ribatti, Stefano Caraffi, Alessandra D’Angelo, Roberto Perris, Domenica Mangieri

**Affiliations:** a Department of Life Sciences, University of ParmaParma, Italy; b Surgical Pathology Unit, University Hospital of ParmaParma, Italy; c Department of Basic Medical Sciences, Neurosciences and Sensory Organs, Section of Human Anatomy and Histology, University of Bari Medical School, National Cancer Institute “Giovanni Paolo II”Bari, Italy; d Centre for Molecular and Translational Oncology (COMT), University of ParmaParma, Italy

**Keywords:** tumour progression, human vulvar leiomyosarcoma, matrix metalloproteinases, angiogenesis, chicken CAM

## Abstract

Gynaecological leiomyosarcoma (gLMS) represent a heterogeneous group of soft tissue sarcoma, characterized by rare incidence, high aggressiveness and propensity to infiltrate secondary organs, poor prognosis and lethality, because of the lack of biological mechanisms that underlying their progression and effective pharmaceutical treatments. This study was focused on some of the aspects of progression and dissemination of a subtype of gLMS namely vulvar LMS (vLMS). We therefore used a vulvar LMS-derived cell line namely SK-LMS-1, coupled with *in vitro* and *in vivo* assays. We observed that SK-LMS-1 cells have a strong invasive capacity *in vitro*, through the activity of matrix metalloproteinases 2 and 9, while *in vivo* these cells induce a strong angiogenic response and disseminate to the chick embryo liver. Therefore, we postulate that metalloproteinases are involved in the spreading behaviour of SK-LMS-1. Further investigations are necessary to better understand the molecular and cellular machinery involved in the progression of this malignancy.

## Introduction

Gynaecological leiomyosarcomas (gLMS) represent a heterogeneous group of soft tissue sarcoma 1–3 of mesenchymal origin 1, that although have a rare incidence, present high aggressiveness, and propensity to infiltrate secondary organs 4–10, poor prognosis and lethality because of the lack of effective treatments 11,12.

While a lot of experimental and clinical studies documented many of the mechanisms involved in the progression of several types of carcinomas 13,14, by contrast, the molecular and cellular mediators involved in progression and dissemination of STSs, and more in detail, in gLMS, remain mostly unknown.

The cleavage of extracellular matrix (ECM) proteins is a pivotal phenomenon in tumour spreading and during metastatic cascade 15,16, facilitating movement of malignant cells and angiogenesis.

In this context, matrix metalloproteinases (MMPs), a group of Zn-dependent enzymes comprising more than 20 members, seem to play a major role 16,17.

In this study, we performed combined *in vitro* and *in vivo* assays on a vulvar LMS cell line, namely SK-LMS-1, to clarify some aspects of progression and dissemination of this lethal disease.

## Materials and methods

### Cell line and conditioned medium

Human vulvar SK-LMS-1 cells were purchased from ATCC (Manassas, VA, USA) and cultured in DMEM (Lonza, Cologne GmbH, Germany) supplemented with 10% foetal bovine serum (FBS; Sigma-Aldrich, Milan, Italy), 2 mM L-Glutamine (Lonza) and antibiotics (100 U of penicillin G and 100 μg/ml of streptomycin sulfate, Lonza). Cell cultures were maintained at 37°C under a humidified 5% CO_2_ atmosphere. Conditioned medium (CM) was prepared from confluent SK-LMS-1 cells culture maintained in DMEM without serum. After 72 hrs the CM was collected, centrifuged at 500 × g for 5 min. at 4°C and filtered through a 0.22 μm pore size membrane.

### Invasion assay

The invasion potential of SK-LMS-1 cells was assessed by using the EnzChek Gelatinase Kit (Life Technologies, Milan, Italy), based on a DQ™ Gelatin fluorescein isothiocyanate. Under the proteolytic action of MMPs, fluorescent peptides were released. The assay was employed as follows: a mixture containing 50 μl of growth factor reduced (GFR) Matrigel (BD Biosciences, Milan, Italy) and 100 μg/ml of DQ™ Gelatin was added to each well of a 96-well plates and allowed to solidify at 37°C for 1 hr. Each well was filled with 1.5 × 10^4^ SK-LMS-1 cells suspended in 100 μl DMEM supplemented with 10% FBS, in presence or absence of metalloproteases (MMPs) inhibitor 1,10-Phenanthroline (1 mM final concentration; Life Technologies). Cultures were monitored at 3 and 72 hrs under an inverted microscope equipped with a CCD camera (DS-Fi2; Nikon Instruments, Florence, Italy) at 100× magnification, and the fluorescence emitted by proteolysis of the FITC-conjugated gelatin was measured using a microplate fluorescence reader (SPECTRAFluor; Tecan Group, Männedorf, Switzerland) at a wavelength range of 485/535 nm. Results were plotted and expressed as arbitrary units of fluorescence (FU).

### Matrigel evasion assay

2 × 10^4^ SK-LMS-1 resuspended in 2 μl of serum-free DMEM were included in a 2 μl mixture of GFR-Matrigel (BD Biosciences) and the above described DQ™ Gelatin (100 μg/ml; Life Technologies). Each drop was seeded at the centre of each well of a 24-well plate and allowed to solidify al 37°C for 30 min. Wells were then filled with DMEM containing 10% FBS in presence or absence of metalloproteases (MMPs) inhibitor 1,10-Phenanthroline (1 mM final concentration; Life Technologies). Cells were monitored daily and images were captured with an inverted fluorescence microscope equipped with a CCD camera (DS-Qi1Mc; Nikon Instruments) at 40× and 200× magnification. Fluorescence emitted by peptides after proteolysis of gelatin was measured using a microplate fluorescence reader (SPECTRAFluor; Tecan Group) at 485/535 nm. Results were plotted and expressed as arbitrary units of fluorescence (FU). Supernatant from each well was collected to prepare CM, as described previously.

### MMPs western blotting analysis

SK-LMS-1 cells inside the Matrigel drops from the evasion assay were collected for Western blot analysis. After 3 and 72 hrs, cells from eight drops were recovered from Matrigel using Recovery Solution (BD Biosciences) according to manufacturer’s instruction and lysed with radioimmunoprecipitation buffer containing a cocktail of protease inhibitors (Roche, Milan, Italy). The protein content was quantified using Bradford reagent (Sigma-Aldrich), and 40 μg aliquots were mixed in Laemmli buffer, boiled at 90°C for 5 min. and loaded on each lane of a precast 4–15% gradient SDS-polyacrylamide gel (Bio-Rad, Segrate (Mi), Italy). After electroforetic resolution, proteins were transferred to a nitrocellulose membrane by a Trans-Blot Turbo Blotting System (BioRad). Blots were then blocked for 1 hr with TBS containing 0.1% Tween-20 (TBST) and 5% skim milk and incubated for 2 hrs with the following primary antibodies, diluted in TBST: 4 μg/ml rabbit polyclonal anti-MMP-2 (ab37150; Abcam, Cambridge, UK); 1:250 mouse monoclonal anti-MMP-9 (ab119906, clone 5G3; Abcam); 1:400 rabbit polyclonal anti-β-actin (A2066; Sigma-Aldrich). Membranes were incubated with specific horseradish peroxidase-conjugated secondary antibodies (1:400; Sigma-Aldrich) at room temperature for 1 hr. Immunocomplexes were visualized using WestarECL system (Cyanagen, Bologna, Italy) and exposure to radiograph film (Kodak, Rochester, NY, USA).

### Angiogenic factors array

Expression of angiogenesis-related proteins contained in the CM derived from the drops was determined with the Human Angiogenesis Array Kit (R&D Systems Ltd., Abingdon, UK), consisting of 55 antibodies against angiogenesis-related proteins spotted on a nitrocellulose membrane. The array was performed following the manufacturer’s instructions using 1 ml of CM or cell supernatant from SK-LMS-1 drops. The data from developed X-ray film were digitalized with a transmission-mode scanner and quantified using ImageJ analysis software (http://rsbweb.nih.gov/ij/). The positive controls were spot couples distributed in the upper left, upper right, and lower left corners of each array kit, whereas the negative controls spot couples were located in the lower right corner of each membrane. The averaged background signal was subtracted and the arrays were calibrated based on the signal strength of the positive controls. The average signal (pixel density) of the pair of duplicated spots representing each angiogenesis protein was determined as above and the corresponding signals on different arrays were compared.

### CAM assay

Fertilized chicken eggs were incubated at 37°C and constant humidity. On day 3 of incubation, a window was opened in the eggs shell after removal of 2–3 ml of albumen so as to detach the developing CAM from the shell, then eggs were sealed with tape and returned to the incubator. On day 8, 5 × 10^5^ cancer cells were labelled with lipophilic dye FastDiO (Life Technologies) and resuspended in 10 μl of GFR-Matrigel (BD Biosciences); 20 μl of total suspension were carefully placed on top of each CAM and eggs were returned to the incubator. Vascular structures were visualized using a stereomicroscope equipped with a CCD camera (DS-Fi2; Nikon Instruments) and pictures were taken daily for 7 days, both in bright field and fluorescence, until embryos were sacrificed on day 15. Blood vessels entering the implant on the focal plane of the CAM were counted at a magnification of 50× using Nikon NIS software. After sacrifice, using a stereomicroscope, samples of CAM (in correspondence of tumour cell graft and as far as possible from the tumour implant) and embryo organs were dissected. Samples were processed for molecular analysis and immunohistochemistry as described below.

### Immunohistochemistry

Samples from CAM and embryo organs were fixed in 4% paraformaldehyde, dehydrated in a graded ethanol series, cleared in xylene and embedded in paraffin. CAM blocks were cut into 8 μm thick sections, whereas embryo internal organs were cut into 6 μm thick sections; all were subjected to immunohistochemical analysis. To discover human cancer cell infiltrates, we used 1 mg/ml rabbit polyclonal anti-human NuMA (Nuclear mitotic apparatus protein; ab97585; Abcam) and 1:50 rabbit monoclonal anti-human mitochondria (MA5-12017, clone MTC02; Thermo Scientific, Waltham, MA, USA) antibodies, diluted in PBS. After 12 hrs of incubation at 4°C, immunodetection was performed by using diaminobenzidine DAKO kit (DAKO, Cernusco sul Naviglio, Milan, Italy) according to the manufacturer’s protocol. Sections were then washed in distilled water, counterstained with haematoxylin (Polysciences, Warrington, PA, USA) and mounted using buffered glycerine. Specific pre-immune serum replacing the primary antibodies served as a negative control. Sections were examined with a Nikon light microscope as described above.

### Alu sequences detection assay

Frozen chick embryo tissues were subjected to DNA extraction using DNeasy Blood and Tissue kit (Qiagen, Milan, Italy) according to the manufacturer’s protocol. PCR was performed on 100 ng of purified DNA using GoTaq® Flexi DNA Polymerase (Promega, Milan, Italy) and human *Alu* specific primers (forward: 5′-ACGCCTGTAATCCCAGCACTT-3′; reverse: 5′-TCGCCCAGGCTGGAGTGCA-3′) 18 at an annealing temperature (Ta) of 60°C. After 20 cycles, the amplification product (244 bp) was electrophoresed on 1.8% agarose gel in Tris-Acetate-EDTA buffer. Images of gels were collected with a gel documentation system (AlphaImager, Alpha Innotech, San Jose, CA, USA).

### Real-time PCR for angiogenic factors

Angiogenic factor transcripts from human tumour cells were detected *via* RT-PCR. Total RNA was extracted from tumour-treated CAM/chick embryo tissues at 4 and 7 days after SK-LMS-1 inoculum using Trizol Reagent (Life Technologies) following the manufacturer’s instructions. For chicken targets, RNA was reverse-transcribed using the QuantiTect Reverse Transcription Kit (Qiagen) and then amplified using the *Power* SYBR® Green PCR Master Mix (Life Technologies). For better sensitivity, human targets were retrotranscribed and amplified in a single step using *Power* SYBR® Green RNA-to-CT™ 1-Step Kit (Life Technologies), according to the manufacturer’s protocol. Both reactions were carried out in a DNA Engine Opticon System (MJ Research Incorporeted, St. Bruno, QC, Canada) with the primer pairs (IDT DNA, Leuven, Belgium) described in Tables1 and 2, using human RPL27 and chicken β-actin as endogenous controls. Each sample was run in triplicate and analysed with the 2^−ΔΔCt^ method.

**Table 1:** Primers for human angiogenic factors analysis

Factor	Forward primer (5′–3′)	Reverse primer (5′–3′)	Ta (°C)
FGF-2	CCGTTACCTGGCTATGAAG	ACTGCCCAGTTCGTTTCAG	65
MMP-2	AATACCATCGAGACCATGC	GTCCAGATCAGGTGTGTAGC	65
MMP-9	CCTTTGGACACGCACG	CCTAGTCCTCAGGGCACT	61
VEGFA	AGAGCAAGACAAGAAAATCCCT	ATCTGGTTCCCGAAACCCT	61
RPL-27	TGGGAAGGTGGTGCTTGT	GGGGTAGCGGTGAATTCC	54

**Table 2:** Primers for chicken angiogenic factors analysis

Factor	Forward primer (5′–3′)	Reverse primer (5′–3′)	Ta (°C)
VEGFA	GAAAGGCCGGTACAAACCA	TTAACTCAAGCTGCCTCGAC	56
β-Actin	CCGCAAATGCTTCTAAACCG	AAAGCCATGCCAATCTCGTC	56

### Statistical analysis

All experiments were performed in triplicate, and data were expressed as mean ± S.D. The statistical correlation between groups was analysed by Student’s *t*-test, where *P* < 0.05 was considered significant.

## Results

### Invasive propensity of SK-LMS-1 requires the production and activation of MMPs

**Figure 1:**
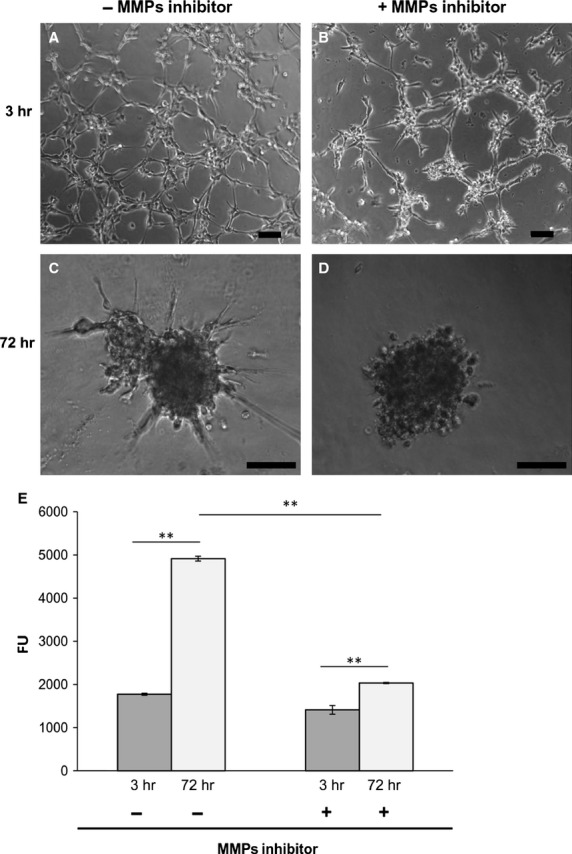
Matrigel invasion assay. (A–D) Phase contrast images of cells on Matrigel layer at indicated time-points; scale bar: 100 μm. (E) Endogenous collagenases activity indirectly measured by fluorescence emitted by DQ™ Gelatin degradation at indicated time-points. Data represent mean of three independent experiments ± S.D. **P < 0.01.

**Figure 2:**
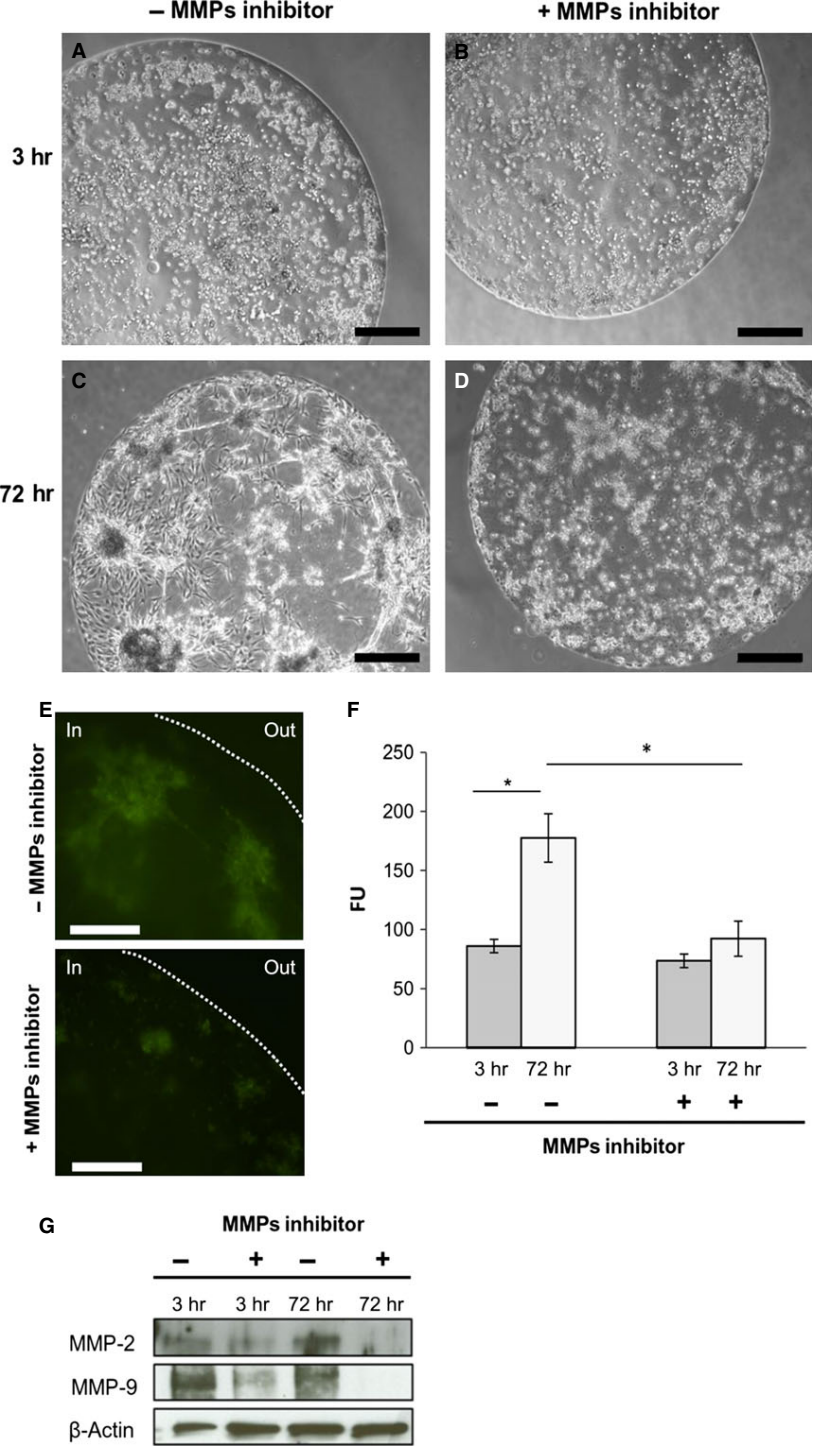
Matrigel drops evasion assay. (A–D) Phase contrast images of drops at indicated time-points; scale bar: 500 μm. (E) Fluorescent images of cells in Matrigel drops 72 hrs after seeding in absence or presence of MMPs inhibitor 1,10-Phenantroline. The dotted line indicates the border of drop; scale bar: 100 μm. (F) Measure of endogenous collagenases activity. Fluorescence was emitted by DQ™ Gelatin degradation at indicated time-points. Data represent mean of three independent experiments ± S.D. (G) Western blot analysis of protein extracted from drops 3 and 72 hrs after seeding. **P < 0.01.

To assess the invasive ability of SK-LMS-1 and to verify if MMPs are implicated in this phenomenon we performed a series of complementary invasion/evasion experiments. During invasion assay (Fig.1), in standard culture conditions, we observed that 3 hrs after seeding in absence of MMPs inhibitor, SK-LMS-1 cells were able to invade the surrounding scaffold, forming well organized tubular-like structures (Fig.1A), by contrast, in presence of MMPs inhibitor, cells were incapable to form a network (Fig.1B). After 72 hrs of culture, the tumour cells arranged in discrete aggregates with many branches, invadopodia-like protrusions penetrating the matrix (Fig.1C) suggesting a clear invasive propensity of the SK-LMS-1. By contrast, adding 1,10-Phenantroline, cells aggregated in rounded, non-invading clusters (Fig.1D). Quantitatively, as shown in Figure1E, after 72 hrs of culture, the MMPs activity of SK-LMS-1 was significantly higher in culture without MMPs inhibitor compared to the control (FU = 4093 ± 56 *versus* FU = 1774 ± 25; *P* < 0.001). In the evasion assay (Fig.2), we found that the cancer cells after 3 and 72 hrs of culture with medium alone already arranged in well organized invadopodia-like structures (Fig.2A and C) when compared with SK-LMS-1 embedded in presence of MMPs inhibitor (Fig.2B and D). Proteolytic activity of MMPs was documented by fluorescence emission in absence (Fig.2E, upper panel) or presence (Fig.2E, lower panel) of MMPs inhibitor. The activity in terms of Fluorescence Units was plotted in Figure2F (FU = 177.5 ± 21 *versus* FU = 86 ± 6; *P* < 0.05). To asses which MMPs were involved, we performed a Western blot analysis on cell lysates derived from drops, and results indicated that MMP-2 and MMP-9 were expressed. In particular, MMP-9 was expressed early, while the expression of MMP-2 arose 72 hrs after seeding (Fig.2G). When the samples were treated with the MMPs inhibitor a reduction in MMPs levels was detectable (Fig.2G).

**Figure 3:**
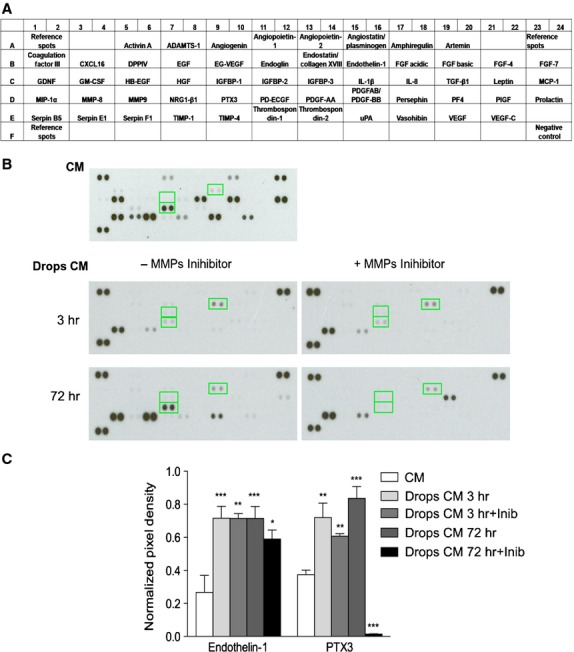
Assessment of angiogenesis-related proteins. (A) Schematic representation of the angiogenic array. (B) Relative expression of 55 different angiogenesis-related proteins are determined from CM or Drops CM at 3 and 72 hrs in absence or presence of MMPs inhibitor 1,10-Phenantroline. All proteins are determined in duplicate and based on optical densitometry of the corresponding spots. The up-regulated expression of proteins in CM and in Drops CM are indicated with green squares. (C) The expression of proteins was quantified and plotted in the reported histogram. IGFBP1 is expressed only in the Drops CM while Endothelin-1 and PTX3 expression is increased in Drops CM in respect to CM. *P < 0.05; **P < 0.01; ***P < 0.001.

To ascertain whether the evasion capacity of SK-LMS-1 was because of the release of pro-invasive/angiogenic molecules, we performed antibody based protein array analysis (Fig.3A). The analysis was performed on CM obtained both from cancer cells seeded in monolayer (CM) and embedded in drops (drops CM). A significant up-regulation of two pro-invasive factors, endothelin-1 and pentraxin 3 (PTX3) was observed in drops CM both after 3 and 72 hrs (Fig.3B, left). Indeed, in drops CM we observed the presence of another pro-invasive protein, insulin-like growth factor binding protein-1 (IGFBP-1; Fig.3B, left). MMPs inhibitor reduced the expression of these proteins (Fig.3B, right). The data were quantified and the normalized pixel density was reported in Figure3C. No expression of MMP-9 was detected in drops CM. This was probably due by the local *in situ* retirement of the gelatinase, degradating the Matrigel embed structure.

### *In vivo* angiogenic and invasive potential

**Figure 4:**
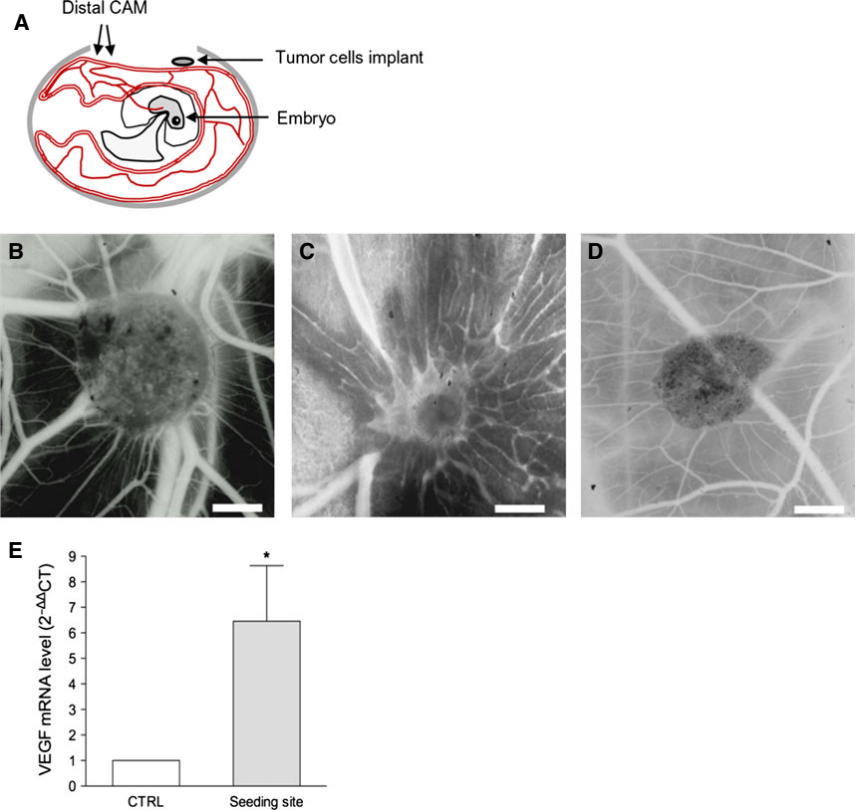
CAM assay. (A) Schematic representation of CAM assay. (B) Stereomicroscopic images of SK-LMS-1 embedded in Matrigel implanted on the CAM. After 4 days numerous allantoic vessels formed radially towards the implant. (C) 200 ng of human FGF-2 resospended in Matrigel was used as positive control. (D) Matrigel alone was used as negative control; scale bar: 1000 μm. (E) Quantification of avian VEGF in tumour-treated CAM samples by RT-PCR. *P < 0.05.

**Figure 5:**
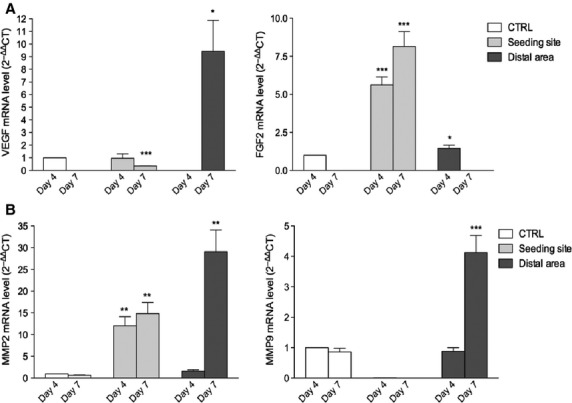
Molecular analysis of human angiogenic factors in SK-LMS-1 treated CAM by RT-PCR. (A) A one step real time PCR system was used to detect the expression of human VEGF (left) and FGF-2 (right) in the SK-LMS-1 CAM seeding site and distal area. (B) The same system was used to asset the expression of both human MMP-9 (left) and MMP-2 (right). Histograms represent means and S.D.s from three independent experiments. *P < 0.05; **P < 0.01; ***P < 0.001.

**Figure 6:**
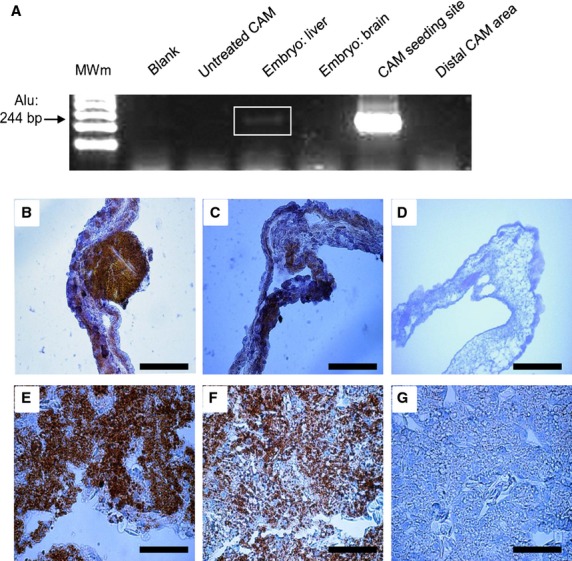
SK-LMS-1 infiltration on CAM and in chick embryo organs. (A) RT-PCR detection of human Alu sequences in different samples of the SK-LMS-1 treated CAM and embryo organs. (B–G) Immunohistochemical analysis of CAM and embryo liver: slides were stained with anti-human mitochondria (B and E) and with anti-human NuMA antibodies (C and F). Negative controls was performed by replacing primary antibodies with specific pre-immune serum (D and G); scale bar: 50 μm.

To study SK-LMS-1 behaviour in term of angiogenic potential, spreading aptitude and metastasis formation *in vivo,* we employed a chicken CAM assay. A schematic representation of the model used is reported in Figure4A. Four days after SK-LMS-1 cells implant, stereoscopic observation documented many neovessels that developed radially towards the tumour implant arranged in a classical ‘spoked-wheel’ pattern (mean number of blood vessels = 35 ± 4; Fig.4B). Angiogenic effect was comparable to that induced by FGF-2, a widely recognized angiogenic factor (mean number of blood vessels = 28 ± 5; Fig.4C). By contrast, only few blood vessels directed towards the implant composed of GFR-Matrigel alone (mean number of blood vessels = 5 ± 1.5; Fig.4D) were identifiable. The angiogenic response was indeed demonstrated by real time PCR showing an up-regulation of the chicken VEGF expression (Fig.4E). To further investigate if the angiogenic response might be related to the secretion of pro-angiogenic factors produced by SK-LMS-1 cells implants, we used a one step real time PCR system to detect human VEGF and FGF-2 in the SK-LMS-1 CAM seeding site and distal area (Fig.5A). We observed that human VEGF is not significantly increased in the seeding site while it is strongly up-regulated after 7 days in the distal area (Fig.5A, left). Expression of human FGF-2 is increased in the site of SK-LMS-1 cells implant 4 and 7 days after engraftment as compared with control (Fig.5A, right). Instead, in the distal areas of the implant a weak increase in FGF-2 expression can be observed only after 4 days (Fig.5A, right). Moreover, we demonstrated that MMP-2 was strongly expressed in the seeding site at 4 and 7 days after implant. Indeed, in the distal area both MMP-2 and MMP-9 were up-regulated 7 days after engraft (Fig.5B). To explore if SK-LMS-1 cells were able to migrate from the site of implant, we performed human Alu sequences analysis by PCR followed by immunohistrochemistry (Fig.6). Results obtained by amplification of Alu sequences (Fig.6A) revealed the presence of human genome in CAM in the site of implant, as expected, and in some embryo organs, such as the liver. Microscopic results confirmed infiltrates of human cells detectable not only in the site of implant (Fig.6B and C) but also in the chicken liver (Fig.6E and F).

## Discussion

The molecular mechanisms underlying the progression and metastasis of vLMS are still unknown.

Here, we have identified MMP-2 and MMP-9 as crucial enzymes in modulating the invasive activity of vLMS SK-LMS-1 cell line; we have shown that vLMS progression and angiogenesis are correlated. In addition we observed a propensity of the SK-LMS-1 tumour cells to colonize internal organs, such as liver, when the cells were implanted on the CAM surface.

In this study, we found that in invasion/migration assays, in standard culture conditions, SK-LMS-1 cells were able to penetrate the gelatinous scaffold in which they were seeded; by contrast, in the presence of MMPs inhibitor 1,10-Phenanthroline, this capacity is significantly reduced. 1,10-Phenatroline is a MMPs inhibitor because of its ability to chelate metals (Zn^2+^, Ca^2+^, Cu^2+^) 19,20. MMPs are frequently implicated in ECM degradation during tumour progression, promoting angiogenesis 15–17, spreading and metastasis 15–17,21,22. Since their activity contributes to the evolution of many tumours 22–26, MMPs have been considered as potential therapeutic targets in cancer treatment 27, and some specific inhibitors have been tested in clinical trials 27,28.

By analysing the molecular composition of CM derived from SK-LMS-1 drops after the evasion assay, we have identified different factors related to the expression and activation of MMPs. At least three molecules were up-regulated: Endothelin-1, PTX3 and IGFBP-1. Endothelin-1 is over-expressed in a number of cancers, influencing different aspects of tumour progression 29, and it induces expression of several MMPs 30. PTX3 has recently been found to affect cell spreading and metastasis formation in breast cancer 31 and other tumours 32. We hypothesize that both Endothelin-1 and PTX3 could influence the expression of MMPs in SK-LMS-1 cells *in vitro*. IGFBP-1 plays a role in promoting the survival and growth of some tumour types 33,34; however, the underlying mechanisms have not been established yet. It is unclear whether the presence of IGFBP-1 in the SK-LMS-1 CM is directly related to MMPs activity.

The use of CAM as a host for SK-LMS-1 cells allowed us to observe two concomitant events: the angiogenic response and the metastatic diffusion of cancer cells, a phenomenon already documented for other tumour types 35–38. Regarding the angiogenic response, we hypothesize that it is mainly under the control of both human and chick VEGF. VEGF is one of the most potent angiogenic factors 39. Since the CAM is a vascularized extraembryonal annex, continuously undergoing blood vessel formation and remodelling is under the control of endogenous angiogenic factors, including VEGF and FGF-2 39,40. In our experimental condition, SK-LMS-1 cells might enable their own growth and spreading by both expressing pro-angiogenic factors and modulating the expression of chick angiogenic factors.

The propensity of SK-LMS-1 cells to spread to distal portions of the CAM mesenchyme as well as to the embryo liver is consistent with the high propensity of vLMS to infiltrate secondary organs 4–10.

Taken together, our results suggest that MMP-2 and MMP-9 are pivotal elements in modulating the invasive activity of vulvar LMS-derived cells both *in vitro* and *in vivo*, and that the chick CAM provides an appropriate microenvironment for the study of human vLMS progression. Further studies are required to elucidate the involvement of the MMPs/angiogenesis axis in the spreading of vLMS and improve our knowledge of potential molecular targets for cancer treatment.
